# Endovascular treatment of Nutcracker syndrome: case report

**DOI:** 10.1590/1677-5449.180135

**Published:** 2019-05-29

**Authors:** Claudia Guimarães Agle, Dejean Sampaio Amorim, Leonardo Cortizo de Almeida, Cesar Amorim Pacheco Neves

**Affiliations:** 1 Faculdade de Tecnologia e Ciências – FTC, Departamento de Medicina, Salvador, BA, Brasil.; 2 Clínica Angiomed, Cirurgia Vascular e Endovascular, Salvador, BA, Brasil.; 3 Hospital Geral Roberto Santos – HGRS, Departamento de Cirurgia Vascular, Salvador, BA, Brasil.

**Keywords:** nutcracker syndrome, nutcracker phenomenon, renal vein entrapment, pelvic congestion syndrome, endovascular procedures

## Abstract

Chronic pelvic pain is a debilitating disease that directly impacts on quality of life and generates costs for health services. Nutcracker Syndrome is an important cause of pelvic pain and consists of a set of signs secondary to compression of the left renal vein, most commonly between the superior mesenteric artery and the aorta. Treatment remains controversial and varies depending on the patient's clinical severity. However, endovascular treatment with renal vein stenting has achieved excellent results. We report the case of a 59 year-old female treated by endovascular repair with a self-expanding nitinol stent. Clinical data, details of the procedure, and follow-up results are presented. Technical success was achieved and there patient reported no postoperative complications. Short-term, there was relief from symptoms and follow-up imaging tests showed improvement.

## INTRODUCTION

Chronic pelvic pain is a debilitating disease with considerable impact on quality of life and productivity. It is defined as non-menstrual or non-cyclic pelvic pain, with duration of at least 6 months, sufficiently intense to interfere in daily activities and requiring clinical or surgical treatment. One of the main causes of chronic pelvic pain is pelvic congestion syndrome (PCS), which has clinical status characterized by varying degrees of pain, dysuria, dysmenorrhea, dyspareunia, and vulvar congestion, very often associated with vulvar varicose veins.^1^ A study by Asciutto et al.^2^ showed that the left gonadal vein (LGV) and right internal iliac vein are the most often involved in PCS (57.7% each).

Laboratory tests often reveal signs of microhematuria, which is possibly associated with nutcracker syndrome (NCS), an anatomic variant in which the superior mesenteric artery (SMA) and the aorta clamp the left renal vein (LRV), causing reflux from this vein and the LGV. According to Robertson & McCuaig,^3^ NCS is a rare disease. Although the exact prevalence of NCS as a cause of PCS has not been quantified, the condition should always be investigated, in view of its importance in the presentation of PCS.

Nutcracker syndrome generally affects women aged from 20 to 40 years, especially multiparous women. The venous reflux provokes varicose veins in the deep and superficial venous pelvic plexus and is responsible for a typical clinical status comprising left flank pain and chronic abdominal pain. In men, the syndrome can manifest in a similar manner and has been described as one of the causes of varicocele.^4^^-^ 6

This article describes a case of NCS, diagnosed and treated with endovascular techniques in a private clinic in the city of Salvador, state of Bahia, Brazil.

## CASE DESCRIPTION

The patient was a 59-year-old female with a history of pain in the left iliac fossa and flank after prolonged standing and dyspareunia with onset 3 years previously. She was free from lower limb edema and other comorbidities. Physical examination was normal other than lower limb telangiectasias. During this period, she consulted with a rheumatologist and gynecologist, who referred her to the vascular surgery service.

**Figure 1:**
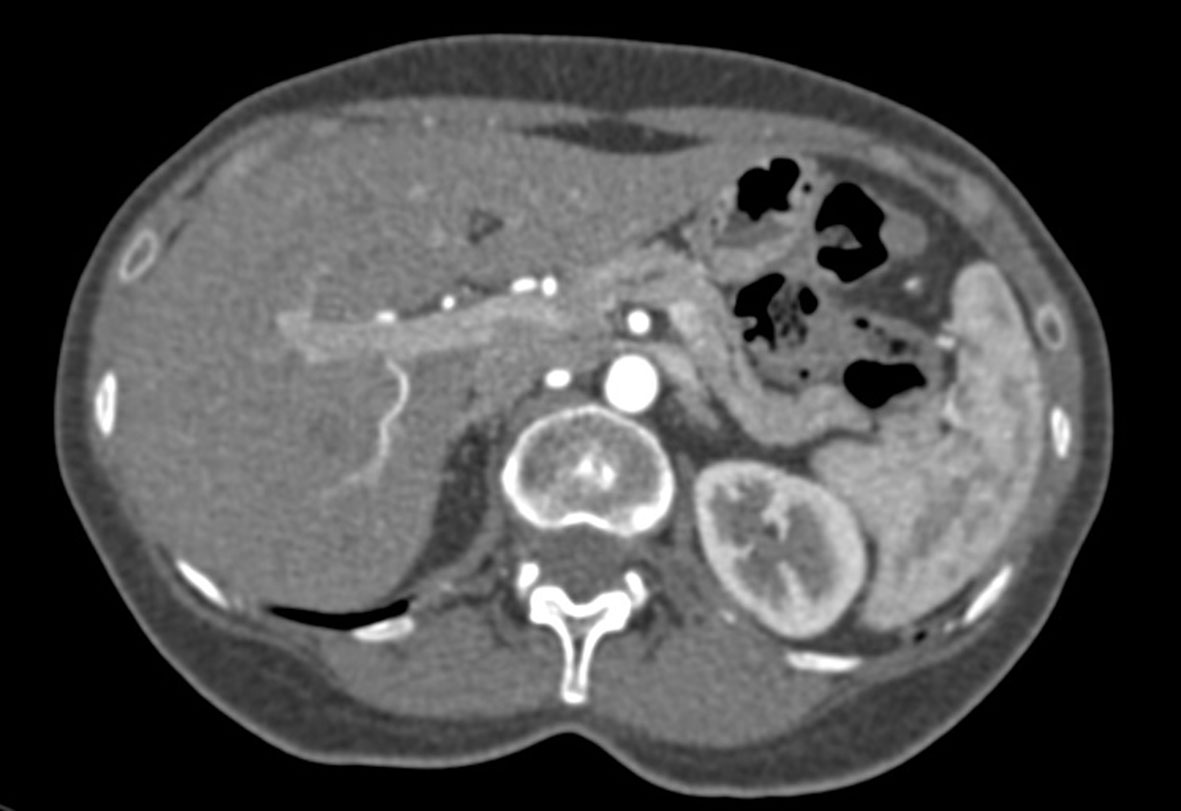
Angiotomography demonstrating compression of the left renal vein.

Transvaginal ultrasonography found adnexal varicose veins on the left. Duplex scan findings of the aorta and renal arteries were normal. Finally, angiotomography revealed a significant reduction of the LRV in the mid third, in the topography of the passage between the SMA and the aorta, associated with ipsilateral adnexal varicose veins, providing evidence of NCS (Figure 1).

**Figure 2:**
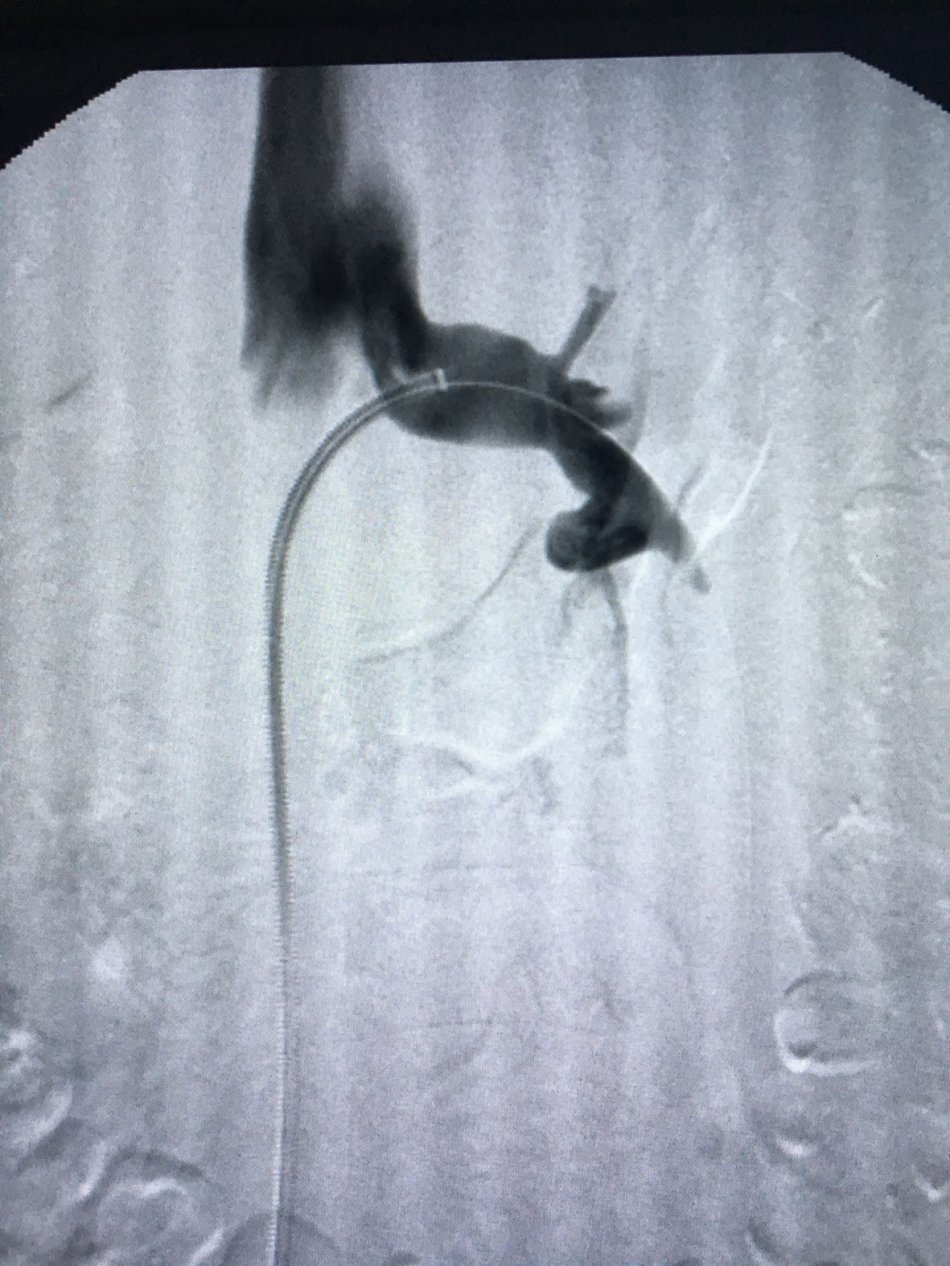
Phlebography showing stenosis of the left renal vein.

After 6 months of conservative treatment, with phlebotonics and analgesics, without resolution, the decision was taken to perform surgical treatment. Repair was performed via endovascular access, after local anesthesia and sedation. The right common femoral vein was punctured and a 6F introducer sheath inserted. The LRV was catheterized with a Cobra 2 5F diagnostic catheter and phlebography was performed. This confirmed critical stenosis of the LRV and pelvic varicose veins on the left, with a discretely dilated gonadal vein (Figures 2 [Fig gf0300]-4). The stenosis pressure gradient was not measured, which could be considered a bias in this case. Treatment consisted of deployment of a Luminex self-expanding nitinol stent (14x40 mm) in the LRV (Figure 5). Additionally, 10 mL of 3% dense polidocanol foam was infused into the adnexal varicose veins. We decided not to embolize the gonadal vein, since it was only discretely dilated.

**Figure 3 gf0300:**
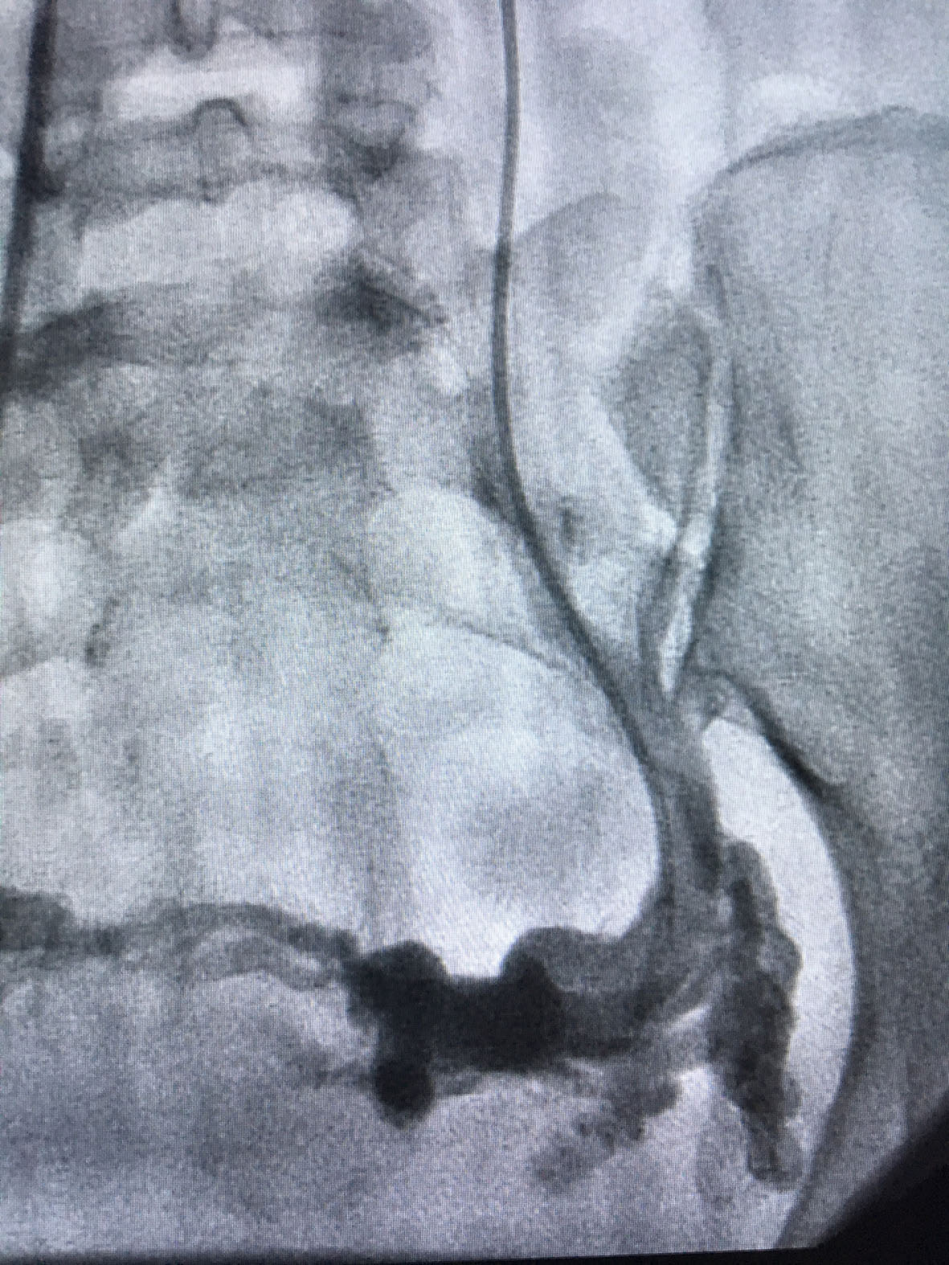
Pelvic varicose veins.

**Figure 4:**
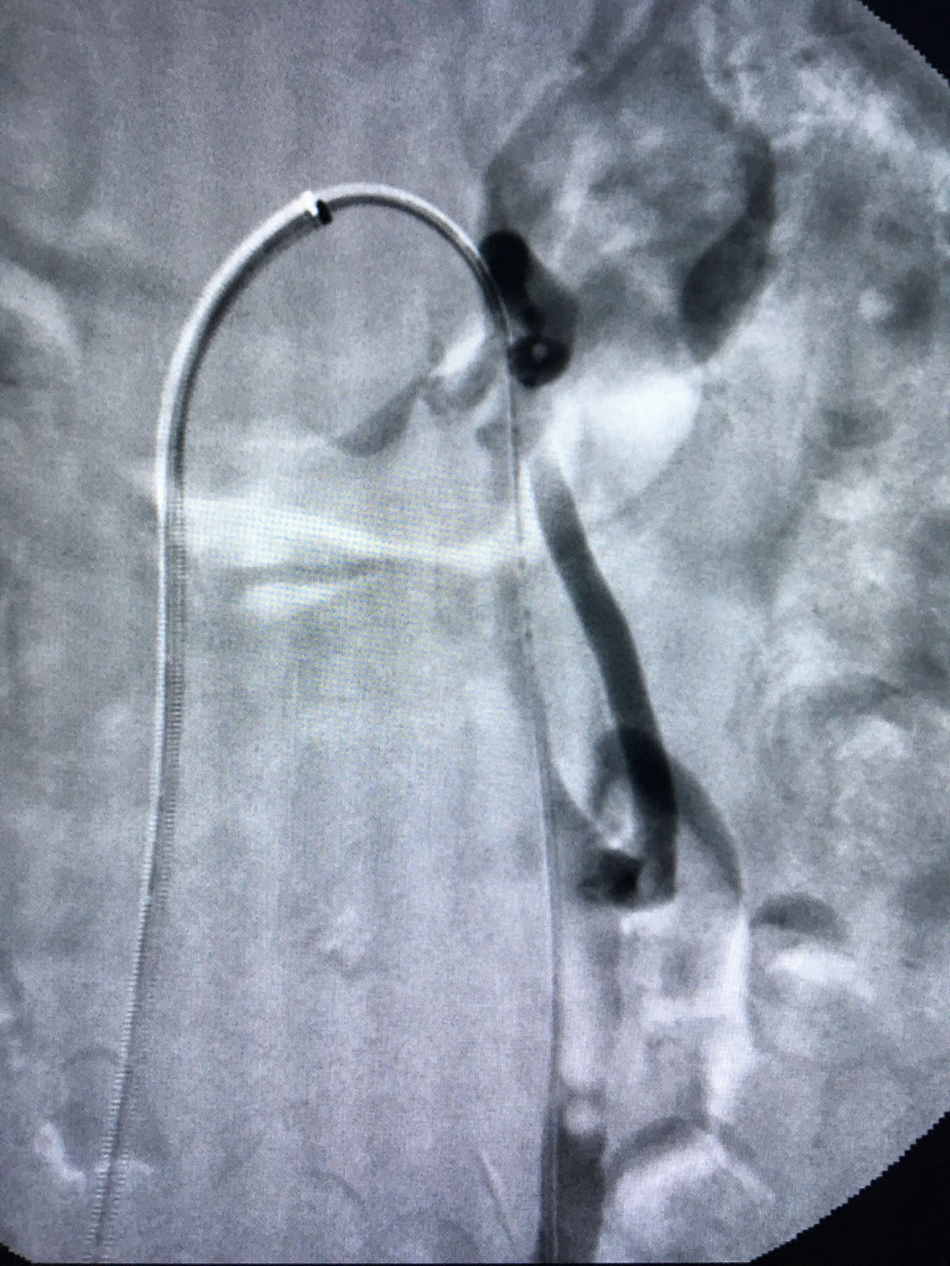
Gonadal vein.

**Figure 5:**
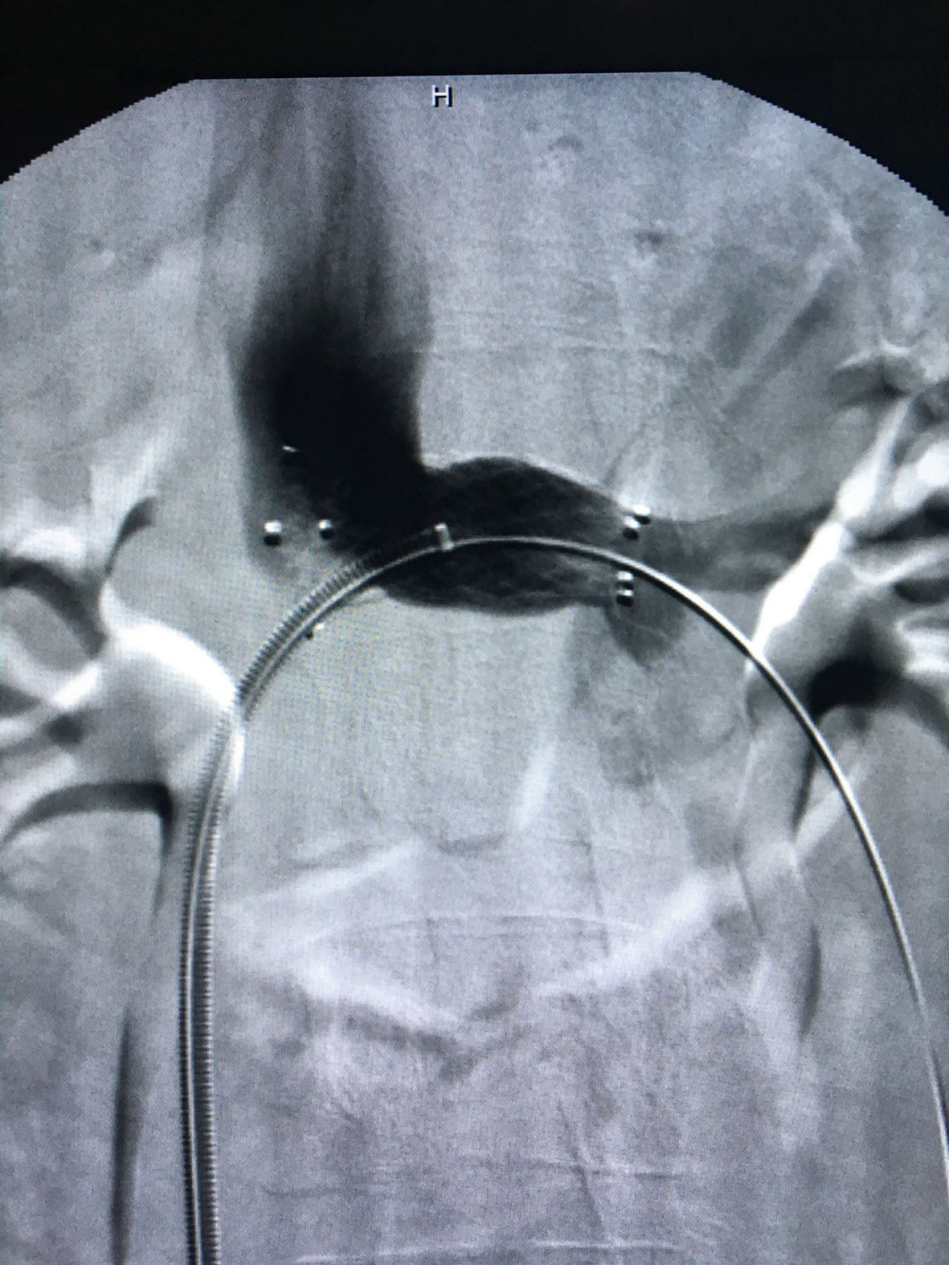
Placement of a stent in the left renal vein.

**Figure 6:**
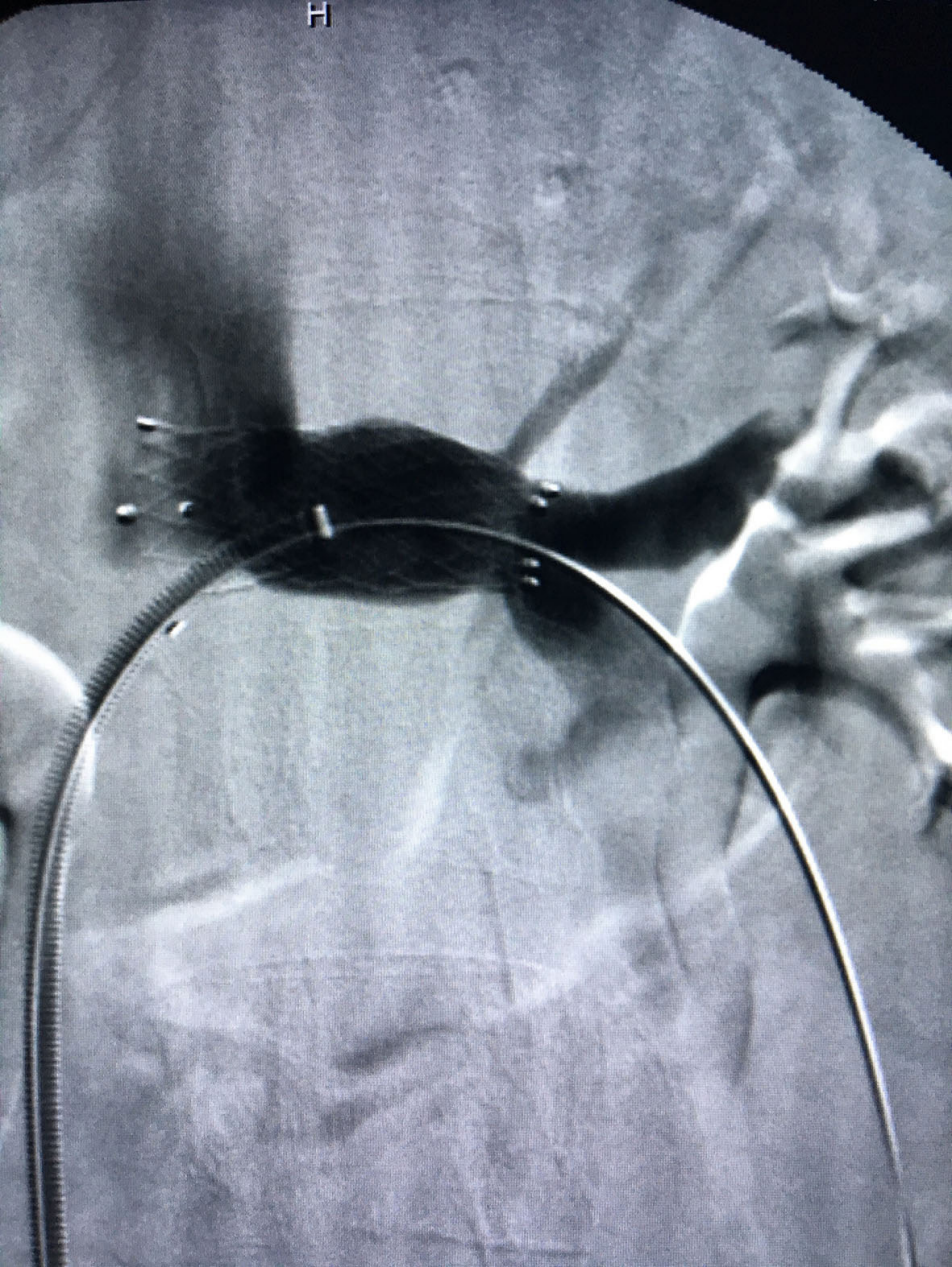
Control phlebography demonstrating complete resolution of left renal vein stenosis.

Control phlebography demonstrated complete resolution of the LRV stenosis (Figure 6).

There were no complications during the surgical procedure or the immediate postoperative period. The patient recovered satisfactorily and was discharged 24 h after the procedure, with immediate relief from pain. She took clopidogrel and acetylsalicylic acid (ASA) for 30 days and then ASA only thereafter.

The patient agreed to publication of this case report and signed an informed consent form.

**Figure 7:**
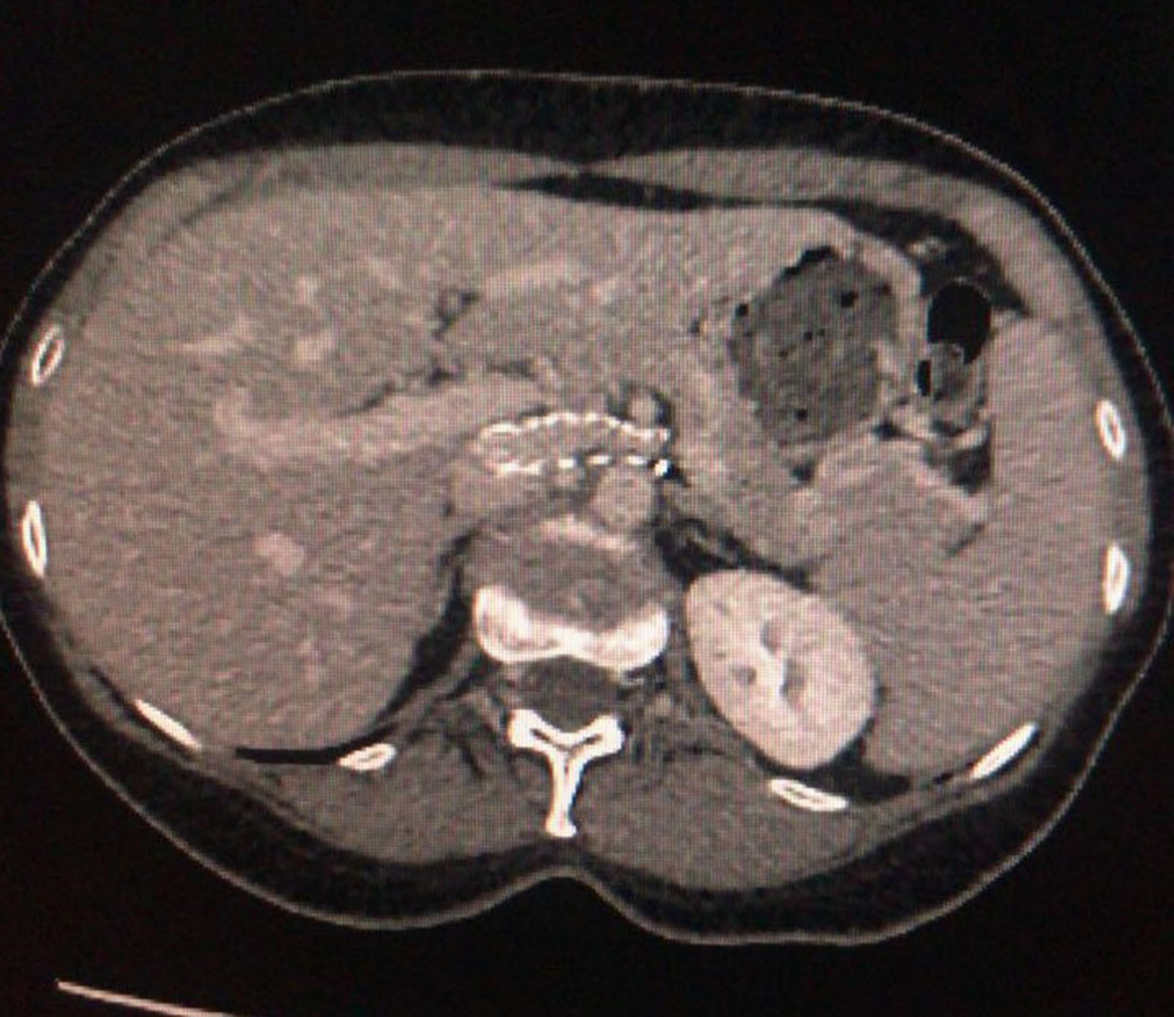
Control tomography showing patent stent 5 months after the procedure.

**Figure 8:**
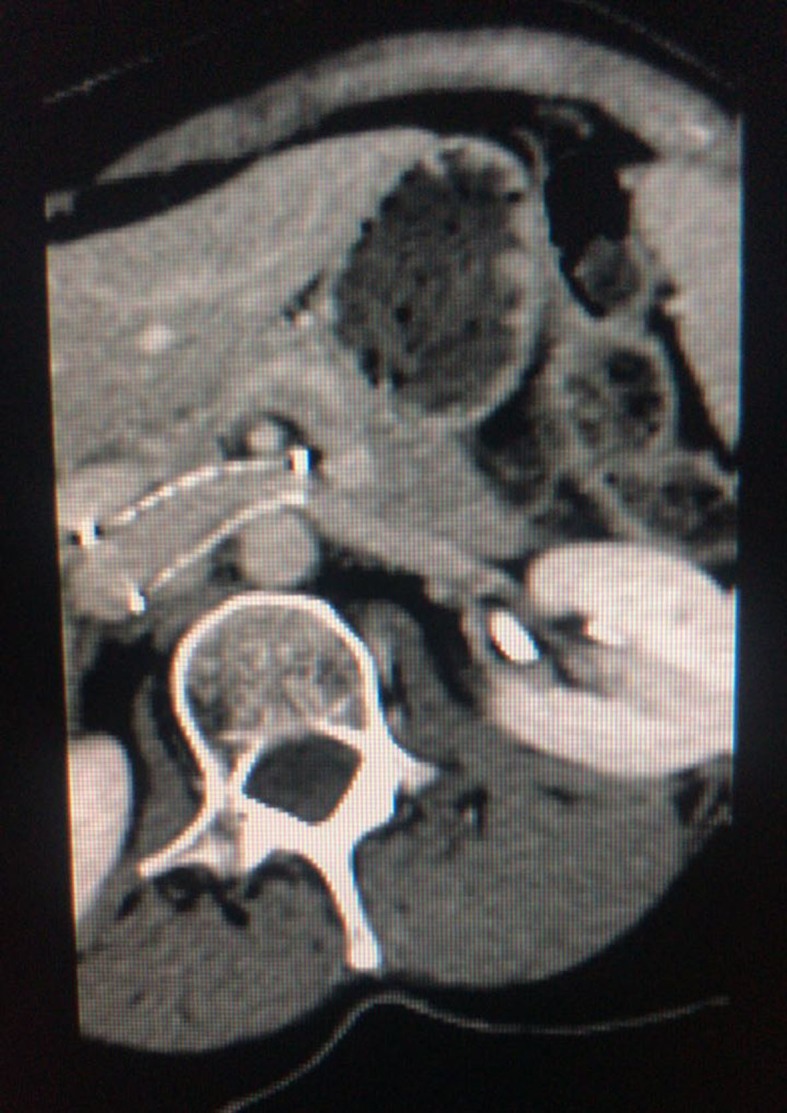
Control tomography showing patent stent 5 months after the procedure.

**Figure 9:**
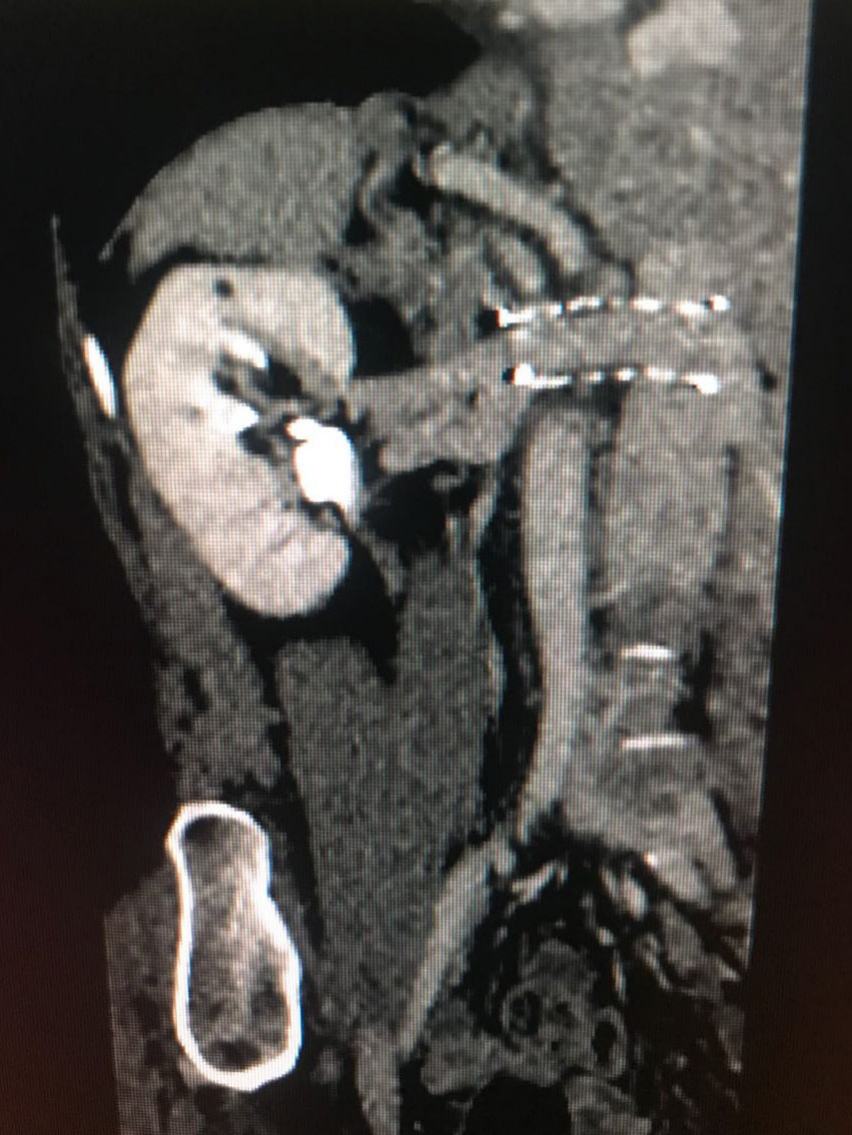
Patent stent on control tomography.

Five months after the endovascular treatment, the patient remained asymptomatic and was in outpatients follow-up at the clinic. During this period, control tomography showed the stent was patent and free from thrombi, with satisfactory correction of the renal compression (Figures 7 and 8). We did not observe residual stenosis, as shown by the control tomography. The stent is fully open and the renal vein free from stenosis (Figure 9).

## DISCUSSION

The nutcracker phenomenon (NCP) consists of compression of the LRV, most often between the aorta and the SMA, with debilitation of blood flow that is frequently accompanied by distension of the hilar portion of the vein. The NCS is the clinical equivalent of the NCP, characterized by a complex of symptoms with substantial variations. The more common examples include hematuria and proteinuria, flank pain, pelvic congestion in female patients, and varicocele in male patients.^7^^,^ 8

The exact prevalence of NCS is unknown, partly because of an absence of definitive diagnostic criteria and the variability of symptomatic presentation. Patients can exhibit the condition at any age from infancy to the seventh decade of life, with peaks in youth (second or third decade, because the rapid increase in height and development of vertebral bodies during puberty can narrow the angle between the aorta and the SMA) and middle-age.^6^^,^ 9 The prevalence of NCS was reported to be greater among women; however, later studies showed that this condition is equally prevalent among both sexes.^6^

Depending on the specific manifestations, NCS may be identified by a number of different medical specialties and, although it is associated with considerable morbidity, diagnosis tends to be difficult and is generally late.^8^ It can be confirmed with the results of imaging exams, including Doppler ultrasonography, tomography, magnetic resonance, phlebography, and intravascular ultrasonography.

Treatment for NCS varies depending on the patient’s clinical severity and is reserved for symptomatic patients only. According to Macedo et al.,^10^ treatment for the syndrome remains a controversial subject, both the choice of the best modality to be used for each patient and the indications for treatment. Options include conservative treatment, open surgery with section of the fibrous ligament between the superior mesenteric artery and the aorta, transposition of the left renal vein, kidney autotransplantation, and even nephrectomy.^6^^,^ 10 ^,^ 11 These techniques can also be performed via laparoscopic access, but experience is limited.^12^

Transposition of the renal vein has been the gold standard for treatment and proven effective. It was employed for the first time in 1982, and appears to be the most common surgical intervention. The procedure is performed using a mid-line transperitoneal approach (mini laparotomy).^11^^,^ 12 Nevertheless, since 1996, endovascular approaches have been gaining in popularity, and have even been recommended as first-line treatment.^13^ Endovascular stent placement is usually preferable to open surgery, because of the long duration of renal congestion, the greater possibility of complications in these cases, and the need for extensive dissection in this type of operation.^10^ Additionally, there is the possibility of simultaneous embolization of the gonadal vein and/or sclerosis with polidocanol directly into the pelvic varicose veins during the procedure. One study comparing 15 patients treated with endovascular methods against five patients treated using open surgery showed that, in the years following the procedure, all of the patients treated with stenting were asymptomatic. Additionally, a minimum of 150 cases of successful endovascular treatment have been described in the medical literature. However, there is still a lack of information from long-term follow-up, which justifies reluctance to employ this treatment with younger patients. Complications include stent migration, intra-stent restenosis, fractures, and venous occlusion.^10^

The Wallstent was the first stent to be used and, according to Macedo et al.,^10^ it remains the first choice among surgeons. With regard to LGV, a literature review^10^ reports relief from symptoms in 56-98% of patients treated with embolization.

The ideal stent should have high radial strength, to eliminate the stenosis, good conformability to adapt to the epithelium of the vessel, and should suffer little length shrinkage, to enable adequate positioning. With our patient, we chose to use a Luminex self-expanding nitinol stent, which offers more precise placement, without risk of shortening like the Wallstent, and we achieved a good result. Embolization of the LGV was unnecessary.

It is important to emphasize that protrusion of the stent into the inferior vena cava should not be considered a complication, since, similar to the technique used by Raju et al.^14^ for treatment of May-Thurner Syndrome, the stent should be placed with considerable protrusion into the inferior vena cava. In our patient, placement of the stent more in the direction of the vena cava and less in the direction of the renal pelvis was primarily because of manipulation of the guidewire, which had been inserted via the gonadal vein. If the stent had been inserted via the renal pelvis, we would not have been able to ensure it was anchored in the exact site, which could have made it difficult to perform a future intervention via the gonadal vein, if such had been necessary.

With relation to anticoagulation and antiplatelet drugs, it should be noted that complications such as thrombosis and stent restenosis can occur, but are rare.^15^ The high blood flow in the renal vein and endogenous urokinase probably contribute to reducing the chances of thrombosis.^13^ When restenosis does occur, it is through myointimal hyperplasia, so an antiplatelet drug is necessary. The recommended regimen consists of 3 days of low molecular weight heparin, 30 days of oral clopidogrel, and 3 months of aspirin.^6^ In our case, we decided to maintain ASA indefinitely.

A high degree of clinical suspicion is needed to diagnose NCS. There are a wide range of treatment possibilities, depending on clinical status and the anatomic characteristics of each case. In this study, the endovascular technique proved safe and effective. The LGV was not embolized, since this conduct does not yet enjoy consensus in the literature.^16^ Costa et al.^17^ described the case of a 24-year-old patient with NCS who was treated with endovascular techniques and without embolization of the LGV, in whom the outcome was disappearance of adnexal varicose veins and complete remission of symptoms. Nevertheless, a recent publication in the Jornal Vascular Brasileiro^10^ supports indication of routine embolization of the gonadal vein combined with stenting of the LRV. In view of this, additional studies would be of use to investigate the necessity of embolization for all cases of NCS, considering the possibility that adnexal veins may revert to their original caliber after correction of reflux and hypertension in the LRV.
