# Supplementary material for: Endovascular treatment of Nutcracker syndrome: case report
Source: J Vasc Bras. 2019 May 29;18:e20180135. doi: 10.1590/1677-5449.180135 (PMC6634940; doi:10.1590/1677-5449.180135)
Supplement: Translation

# Translation: Tratamento endovascular da síndrome de quebra-nozes: relato de caso

**Authors:** Claudia Guimarães Agle, Dejean Sampaio Amorim, Leonardo Cortizo de Almeida, Cesar Amorim Pacheco Neves

**Affiliations:** 1 Faculdade de Tecnologia e Ciências – FTC, Departamento de Medicina, Salvador, BA, Brasil.; 2 Clínica Angiomed, Cirurgia Vascular e Endovascular, Salvador, BA, Brasil.; 3 Hospital Geral Roberto Santos – HGRS, Departamento de Cirurgia Vascular, Salvador, BA, Brasil.

**Keywords:** síndrome de quebra-nozes, fenômeno de quebra-nozes, encarceramento da veia renal, síndrome da congestão pélvica, procedimentos endovasculares

## Abstract

A dor pélvica crônica é uma doença debilitante, com impacto na qualidade de vida e custos para os serviços de saúde. A síndrome de quebra-nozes é uma importante causa dessa dor, e se refere a um conjunto de sinais secundários à compressão da veia renal esquerda, mais comumente entre a artéria mesentérica superior e a aorta. Seu tratamento ainda permanece controverso e varia de acordo com a gravidade clínica do paciente. Contudo, a técnica endovascular com implante de stent em veia renal tem obtido excelentes resultados. Relatamos um caso de uma paciente de 59 anos submetida a correção endovascular com stent autoexpansível de nitinol. São apresentados dados clínicos, detalhes do procedimento e resultados do acompanhamento dessa paciente. O sucesso técnico foi obtido e não houve relato de complicações pós-operatórias. Pôde-se observar alívio dos sintomas e melhora nos exames de imagem realizados no acompanhamento de curto prazo.

## INTRODUÇÃO

A dor pélvica crônica é uma doença debilitante, com grande impacto na qualidade de vida e na produtividade. Ela é definida como dor pélvica não menstrual ou não cíclica, com duração de pelo menos 6 meses, suficientemente intensa para interferir em atividades habituais e que necessita de tratamento clínico ou cirúrgico. Entre as causas de dor pélvica crônica, destaca-se a síndrome de congestão pélvica (SCP), com quadro clínico caracterizado por graus variados de dor, disúria, dismenorreia, dispareunia e congestão vulvar, muitas vezes acompanhada de varizes vulvares^1^. O estudo de Asciutto et al.^2^ revelou que as veias gonadal esquerda (VGE) e ilíaca interna direita são as mais acometidas na SCP (57,7% cada).

Exames laboratoriais para esses casos frequentemente mostram sinais de microhematúria, possivelmente associada à síndrome do quebra-nozes (SQN), uma variação anatômica na qual a artéria mesentérica superior (AMS) e a aorta realizam um clampeamento da veia renal esquerda (VRE), com consequente refluxo dessa veia e da VGE. Segundo Robertson & McCuaig^3^, a SQN é uma doença rara. Apesar de não haver uma quantificação exata sobre a prevalência da SQN como causa de SCP, essa condição deve ser sempre investigada, diante da sua importância no quadro de SCP.

A SQN geralmente afeta as mulheres de idade entre 20 e 40 anos, especialmente mulheres multíparas. O refluxo venoso provoca varizes do plexo venoso pélvico profundo e superficial e é responsável por um quadro clínico típico dor em flanco esquerdo e dor abdominal crônica. Nos homens, essa síndrome pode se manifestar de forma semelhante, sendo uma das causas descritas de varicocele^4^^-^ 6.

Este trabalho relata um caso de SQN, diagnosticado e tratado por meio da técnica endovascular em uma clínica particular na cidade de Salvador (BA).

## DESCRIÇÃO DO CASO

Paciente do sexo feminino, 59 anos, com história de dor em fossa ilíaca e flanco esquerdos após ortostatismo prolongado e dispareunia há 3 anos. Negava edema de membros inferiores e comorbidades. O exame físico revelou-se normal, apenas com telangiectasias em membros inferiores. Durante esse tempo, se consultou com reumatologista e ginecologista, que a encaminhou para o Serviço de Cirurgia Vascular.

Foi realizada uma ultrassonografia transvaginal que identificou varizes anexiais à esquerda. O *duplex scan* de aorta e artérias renais mostrou-se sem alterações. Por fim, a angiotomografia revelou uma redução significativa da VRE no terço médio, na topografia da sua passagem entre a AMS e a aorta, associada a varizes anexiais homolaterais evidenciando a SQN (Figura 1).

**Figura 1:** Angiotomografia demonstrando a compressão da veia renal esquerda.

Após 6 meses de terapia conservadora com uso de flebotônicos e analgésicos sem resolução do quadro, optou-se pelo tratamento cirúrgico. A correção foi feita por via endovascular, após anestesia local e sedação. Foi feita a punção da veia femoral comum direita e colocada a bainha introdutora 6F. Foi realizada cateterização da VRE com cateter diagnóstico Cobra 2 5F e flebografia. Esta confirmou a estenose crítica de VRE e varizes pélvicas à esquerda, com veia gonadal discretamente dilatada (Figuras 2 [Fig gf03]-4). Não foi medido o gradiente de pressão da estenose, o que pode ser um viés do caso. O tratamento foi feito com implante de stent autoexpansível de nitinol (14x40 mm) Luminex na VRE (Figura 5). Também foi realizada infusão de 10 mL de polidocanol a 3% em forma de espuma densa nas varizes anexiais. Optamos por não embolizar a veia gonadal, já que esta apresentava-se apenas discretamente dilatada.

**Figura 2:** Flebografia evidenciando estenose de veia renal esquerda.

**Figura 3 gf03:** Varizes pélvicas.

**Figura 4:** Veia gonadal.

**Figura 5:** Implantação de stent em veia renal esquerda.

A flebografia de controle demonstrou resolução completa da estenose de VRE (Figura 6).

**Figura 6:** Flebografia de controle demonstrando resolução completa da estenose de veia renal esquerda.

Não houve intercorrência durante o procedimento cirúrgico ou no pós-operatório imediato. A paciente evoluiu satisfatoriamente e recebeu alta 24 h após o procedimento, com melhora imediata da dor. Fez uso de clopidogrel e ácido acetilsalicílico (AAS) durante 30 dias e manteve apenas o AAS após o término desse período.

A paciente concordou em participar do relato de caso mediante assinatura do termo de consentimento livre e esclarecido.

Após 5 meses do tratamento endovascular, a paciente mantinha-se assintomática em acompanhamento ambulatorial no serviço. Nesse período, foi realizada uma tomografia de controle, que evidenciou stent pérvio e sem trombos, com correção satisfatória da compressão renal (Figuras 7 e 8). Não visualizamos estenose residual, conforme mostra a tomografia de controle. O stent está totalmente aberto e a veia renal sem estreitamento (Figura 9).

**Figura 7:** Tomografia de controle mostrando stent pérvio 5 meses após o procedimento.

**Figura 8:** Tomografia de controle mostrando stent pérvio 5 meses após o procedimento.

**Figura 9:** Stent pérvio em tomografia de controle.

## DISCUSSÃO

O fenômeno do quebra-nozes (FQN) refere-se à compressão da VRE, mais comumente entre a aorta e a AMS, com fluxo sanguíneo debilitado frequentemente acompanhado por distensão da porção hilar da veia. A SQN é o equivalente clínico do FQN, caracterizada por um complexo de sintomas com variações substanciais. Exemplos mais comuns incluem hematúria e proteinúria, dor no flanco, congestão pélvica em pacientes do sexo feminino e varicocele em pacientes do sexo masculino^7^^,^ 8.

A prevalência exata da SQN é desconhecida, em parte devido à ausência de critérios diagnósticos definitivos e à variabilidade na apresentação sintomática. Os pacientes podem apresentar o quadro em qualquer idade desde a infância até a sétima década de vida, com picos culminando em jovens (segunda ou terceira década, já que o rápido aumento de altura e desenvolvimento dos corpos vertebrais na puberdade pode resultar em um estreitamento do ângulo entre a aorta e a AMS) e em adultos de meia-idade^6^^,^ 9. A prevalência da SQN foi relatada como maior em mulheres; no entanto, estudos posteriores mostraram que essa condição é igualmente prevalente entre os sexos^6^.

Dependendo de manifestações específicas, a SQN pode ser encontrada por diferentes especialidades médicas e, embora seja associada a considerável morbidade, seu diagnóstico costuma ser difícil e geralmente atrasado^8^. Este pode ser confirmado por meio de resultados de imagem, incluindo Doppler, tomografia, ressonância magnética, flebografia e ultrassonografia intravascular.

O tratamento da SQN varia de acordo com a gravidade clínica do paciente e está reservado apenas para pacientes sintomáticos. Segundo Macedo et al.^10^, o tratamento da síndrome ainda permanece um tópico controverso, tanto na escolha da melhor modalidade a ser utilizada para cada paciente quanto nas indicações para tratamento. As opções incluem tratamento conservador, cirurgia aberta com secção do ligamento fibroso entre a artéria mesentérica superior e a aorta, transposição da veia renal esquerda, autotransplante renal e até nefrectomia^6^^,^ 10 ^,^ 11. Essas técnicas podem também ser realizadas por via laparoscópica, mas as experiências ainda são limitadas^12^.

A transposição de veia renal tem sido o padrão-ouro para tratamento, e se mostrou eficaz. Foi aplicada pela primeira vez em 1982, e parece ser a intervenção cirúrgica mais frequente. O procedimento é executado utilizando uma abordagem transperitoneal na linha média (mini laparotomia)^11^^,^ 12.Contudo, desde 1996 a abordagem endovascular vem ganhando força, e já foi até recomendada como primeira opção de tratamento^13^. A colocação de stent endovascular é usualmente preferível à cirurgia aberta, devido ao longo período de congestão renal, por conta da maior possibilidade de complicações nesses casos, e à necessidade de extensa dissecção presentes nesse tipo de operação^10^. Além disso, há a possibilidade de realização simultânea de embolização da veia gonadal e/ou esclerose com polidocanol diretamente nas varizes pélvicas durante o procedimento. Um estudo realizado comparando 15 pacientes tratados por método endovascular com cinco pacientes tratados por meio de cirurgia aberta mostrou que, nos anos seguintes ao procedimento, todos os pacientes tratados com stent estavam assintomáticos. Além disso, já foram relatados na literatura médica no mínimo 150 casos bem-sucedidos de tratamento endovascular. Entretanto, ainda faltam informações sobre o acompanhamento a longo prazo, o que justifica o receio em usar o tratamento em pacientes jovens. Complicações incluem migração do stent, reestenose intra-stent, fraturas e oclusão venosa^10^.

O Wallstent foi o primeiro stent utilizado e, segundo Macedo et al.^10^, ainda permanece como preferência entre os cirurgiões. Quanto à VGE, uma revisão de literatura^10^ relata alívio dos sintomas em 56-98% dos pacientes por meio da embolização.

O stent ideal deve ter alta força radial para abolir a estenose, boa conformabilidade para se adaptar ao epitélio do vaso, e pouco encolhimento no comprimento para permitir um posicionamento adequado. Na nossa paciente, optamos por usar o stent autoexpansível de nitinol Luminex, que é mais preciso na sua colocação, sem risco de encurtamento como o Wallstent, e obtivemos um bom resultado. Não foi necessária a embolização da VGE.

É importante destacar que não se deve considerar a protrusão do stent na veia cava inferior como uma complicação, pois, de forma semelhante à técnica usada por Raju et al.^14^ no tratamento da síndrome de May-Thurner, o stent deve ser posicionado com uma larga protrusão na veia cava inferior. Na nossa paciente, a colocação do stent mais em direção à veia cava e menos em direção à pelve renal se deu principalmente devido à manipulação do fio-guia, que tinha sido introduzido pela veia gonadal. Caso o stent tivesse sido inserido através da pelve renal, não poderíamos assegurar a sua fixação no local exato, o que poderia dificultar uma futura intervenção pela veia gonadal, caso esta viesse a ser necessária.

Com relação à anticoagulação e à antiagregação, vale ressaltar que complicações como trombose e reestenose do stent podem acontecer, mas são raras^15^. O alto fluxo sanguíneo na veia renal e a uroquinase endógena provavelmente contribuem para o decréscimo nas chances de trombose^13^. Quanto à reestenose, esta ocorre por hiperplasia miointimal, portanto se faz necessário o uso do antiagregante plaquetário. O esquema recomendado consiste em 3 dias de heparina de baixo peso molecular, 30 dias de clopidogrel oral, e 3 meses de aspirina^6^. No nosso caso, optamos por manter o AAS perenemente.

A SQN requer um alto índice de suspeição para seu diagnóstico. O tratamento passa por uma ampla gama de possibilidades, dependendo do quadro clínico e das características anatômicas de cada caso. Neste estudo, a técnica endovascular se mostrou segura e eficaz. A embolização da VGE não foi realizada, já que essa conduta ainda não é consenso na literatura^16^. Costa et al.^17^ relataram o caso de uma paciente de 24 anos, portadora de SQN, tratada por técnica endovascular sem embolização de VGE que teve como desfecho o desaparecimento das varizes anexiais e a remissão completa dos sintomas. Contudo, publicação recente do Jornal Vascular Brasileiro^10^ fortalece a indicação de embolização da veia gonadal de rotina associada ao implante de stent na VRE. Diante disso, novos estudos podem auxiliar a investigar sobre a necessidade de embolização para todos os casos de SQN, considerando a possibilidade de retorno das veias anexiais ao seu calibre original após correção do refluxo e da hipertensão da VRE.
